# Differential gene expression between hygienic and non-hygienic honeybee (*Apis mellifera* L.) hives

**DOI:** 10.1186/s12864-015-1714-y

**Published:** 2015-07-07

**Authors:** Sébastien Boutin, Mohamed Alburaki, Pierre-Luc Mercier, Pierre Giovenazzo, Nicolas Derome

**Affiliations:** Institut de Biologie Intégrative et des Systèmes (IBIS), Université Laval, Pavillon Charles-Eugène Marchand, bureau 1253, 1030, Avenue de la Médecine, G1V 0A6 Québec, QC Canada; Département de biologie, Faculté des sciences et de genie, Université Laval, Québec, Canada; Entomology and Plant Pathology Department, West TN Research and Education Center, The University of Tennessee, 605 Airways Blvd, Jackson, TN 38301 USA

**Keywords:** Honeybee, Gene expression, Hygienic behavior, Cytochrome P450 and QTL regions

## Abstract

**Background:**

Hygienic behavior is a complex, genetically-based quantitative trait that serves as a key defense mechanism against parasites and diseases in *Apis mellifera*. Yet, the genomic basis and functional pathways involved in the initiation of this behavior are still unclear. Deciphering the genomic basis of hygienic behavior is a prerequisite to developing an extensive repertoire of genetic markers associated to the performance level of this quantitative trait. To fill this knowledge gap, we performed an RNA-seq on brain samples of 25 honeybees per hives from five hygienic and three non-hygienic hives.

**Results:**

This analysis revealed that a limited number of functional genes are involved in honeybee hygienic behavior. The genes identified, and especially their location in the honeybee genome, are consistent with previous findings. Indeed, the genomic sequences of most differentially expressed genes were found on the majority of the QTL regions associated to the hygienic behavior described in previous studies. According to the Gene Ontology annotation, 15 genes are linked to the GO-terms DNA or nucleotide binding, indicating a possible role of these genes in transcription regulation. Furthermore, GO-category enrichment analysis revealed that electron carrier activity is over-represented, involving only genes belonging to the cytochrome P450. Cytochrome P450 enzymes’ overexpression can be explained by a disturbance in the regulation of expression induced by changes in transcription regulation or sensitivity to xenobiotics. Over-expressed cytochrome P450 enzymes could potentially degrade the odorant pheromones or chemicals that normally signal the presence of a diseased brood before activation of the removal process thereby inhibit hygienic behavior.

**Conclusions:**

These findings improve our understanding on the genetics basis of the hygienic behavior. Our results show that hygienic behavior relies on a limited set of genes linked to different regulation patterns (expression level and biological processes) associated with an over-expression of cytochrome P450 genes.

## Background

The honeybee (*Apis mellifera*) is a valued resource for both mankind and the global environment. Honey is an important food product internationally, but pollination is by far the honeybee’s most valuable contribution [1]. Bees contribute to almost 90 % of crop pollination around the world [2, 3]. In Canada, beekeepers need to store their hives to protect them from difficult wintering conditions. This storage seems to increase the colony sensitivity to infections, which is translated into a greater mortality of bees during the winter [4, 5].

However, the mechanisms involved in this decline of population linked to winter’s mortality remain unclear. Some studies put forward the use of chemicals pesticides, including acaricides, which are detected inside the hives [6–8]. Sublethal exposures to chemicals like neonicotinoid pesticides lead to a disturbance of the behavior of foragers that failed to return to the hives [9]. On another point of view, a higher pathogen incidence can be responsible of the decline observed. That is why some research pointed to the impact of different pathogens [10–14]. Overall, it appears that some factors (pathogen outbreaks, pesticides…) or combinations of factors compromise the immunity of bees, and alter their behavior [6–15].

In honeybees, immunity operates on different levels [16]. Individual immunity encompasses behavioral (auto-grooming), mechanical, physiological and immunological defenses [17–19]. Pairwise defenses include allo-grooming and a colony-wide behavioral mechanism called hygienic behavior [20], a type of nest-cleaning behavior. Nurse bees in response to diseased or dead brood exercise this collective mechanism. Hygienic behaviour is performed by younger bees (<27 days old) and mainly by middle age bees (15–17,5 days old) [21, 22]. This cleaning is accomplished by two different actions. First, the nurses uncap the brood, which is operculated by wax (uncapping), and secondly, they remove the pupae from the brood cell (removal).

This hygienic behavior was first described in 1937 by Park, but its genetic basis was first suggested by Rothenbuhler *et al.* (1964)*,* who proposed a two loci model to explain hygienic behavior inheritance [22–28]. Since then, this behavior has been recognized as an example of the influence of mendelian inherited genes on behavior. One locus (u) was thought to be involved in uncapping and the other (r) in removal. The homozygote for one of the loci should either uncap (uu) or remove (rr). Later, a three loci model was developed to better fit the original data [29]. Recently, four studies based on molecular techniques (RAPD and SNP) found respectively seven, six, nine and two QTLs associated with hygienic behavior [30–33]. These results suggest that the genetic basis of hygienic behavior is more complex than previously thought.

A diseased brood detection threshold is determined by how quickly a nurse can detect and initiate the diseased brood removal process. This detection seems to be influenced by the olfactory capabilities of nurse bees [34–36]. Furthermore, it seems that all worker bees show various levels of hygienic behavior and its effectiveness is linked to the speed of execution. Brain gene expression is closely related to behavioral status in honeybees [37]. Therefore, in order to ensure identification of a reliable signal correlating both gene expression and hygienic behavior, we examined brain tissue from nurse bees of colonies that were the most contrasted in terms of the phenotypic trait of interest. This strategy has been proven to be valuable for detecting candidate genes [37]. In our study, we analyzed the transcriptomic profiles of 13 managed honeybee colonies. The objective was to investigate and compare differential gene expression between hygienic and non-hygienic lines in order to identify genes involved in hygienic behavior. Ultimately, the goal was to provide functional genetic markers for SNP analysis in order to develop useful genomic tools for honeybee selection programs.

## Results

In 2012, the 13 hives were evaluated for hygienic behavior using the freeze-killed brood assay [38]. Data from a previous evaluation in 2011, performed on the same colonies, was also available (Table 1). Comparison of the two evaluations showed that hygienic behavior varied between years. To avoid any bias, we chose to classify the colonies as hygienic or not based on the 2012 evaluation. A wide range of hygienic behaviors was observed during our experiment (Table 1). In 2012, three colonies were classified as non-hygienic (removal of dead brood < 50 %), five exhibited intermediate behavior and five were highly hygienic (removal of dead brood > 90 %). Extreme behaviors were selected for the transcriptome analysis in order to increase the detection power of DEG.

**Table 1:** Hygienic evaluation of the honeybee colonies studied. Hygienic behavior is calculated as a percentage based on the number of dead brood removed in 24 h. Brood were killed by liquid nitrogen

Colony number	Hygienic status	Dead brood removal in 2011 (%)	Dead brood removal in 2012 (%)	Mean dead brood removal in 2011 and 2012 (%)
511	Intermediate	97	86	91.5
529	Intermediate	37.29	71	54.1
538	Non-hygienic	39.5	47.1	43.3
539	Intermediate	46.06	83.6	64.8
551	Intermediate	56.52	75.8	66.1
562	Non-hygienic	58.42	31.1	44.7
564	Non-hygienic	57.5	56.7	57.1
571	Hygienic	92	97.4	94.1
573	Hygienic	97.22	90.4	93.8
586	Hygienic	100	100	100
588	Hygienic	98.13	99	98.5
589	Hygienic	95.76	100	97.8
594	Intermediate	100	87	93.5

A total of 293 296 626 reads were sequenced for the eight colonies most distinct in terms of hygienic behavior (Table 2). The depth of sequencing among the different samples was homogeneous (36 662 078.3 ± 5 670 213.22 reads). The highest quality dataset was for colony 562 (88.95 % of clean reads) and the lowest for colony 586 (87.74 %). We observed few variations in quality among samples (88.47 % ± 0.4), (Table 2). Further, a high number of the clean reads were assembled and mapped to the reference genome of *A. mellifera* (85.56 % ± 0.87) (Table 2). Unmapped reads were not retained in the analysis.

**Table 2:** Statistical description of the sequencing data. Good quality reads were pairs of reads with a phred score value higher than 20. Reads mapped were reads actually mapped to the reference genome of *Apis mellifera*

Colony number	Hygienic status	Raw count	Good quality paired reads (%)	Reads mapped (%)
538	Non-hygienic	43 478 686	38 458 034 (88.45)	33 150 917 (86.2)
562	Non-hygienic	44 198 908	39 317 018 (88.95)	33 189 836 (84.42)
564	Non-hygienic	30 505 102	26 980 714 (88.45)	22 822 890 (84.59)
571	Hygienic	31 143 818	27 466 482 (88.19)	23 619 801 (85.99)
573	Hygienic	36 520 114	32 405 720 (88.73)	27 663 779 (85.37)
586	Hygienic	41 279 310	36 218 642 (87.74)	31 306 577 (86.44)
588	Hygienic	34 888 770	31 017 208 (88.90)	26 304 651 (84.81)
589	Hygienic	31 281 918	27 630 536 (88.33)	23 938 387 (86.64)

**Fig. 1:**
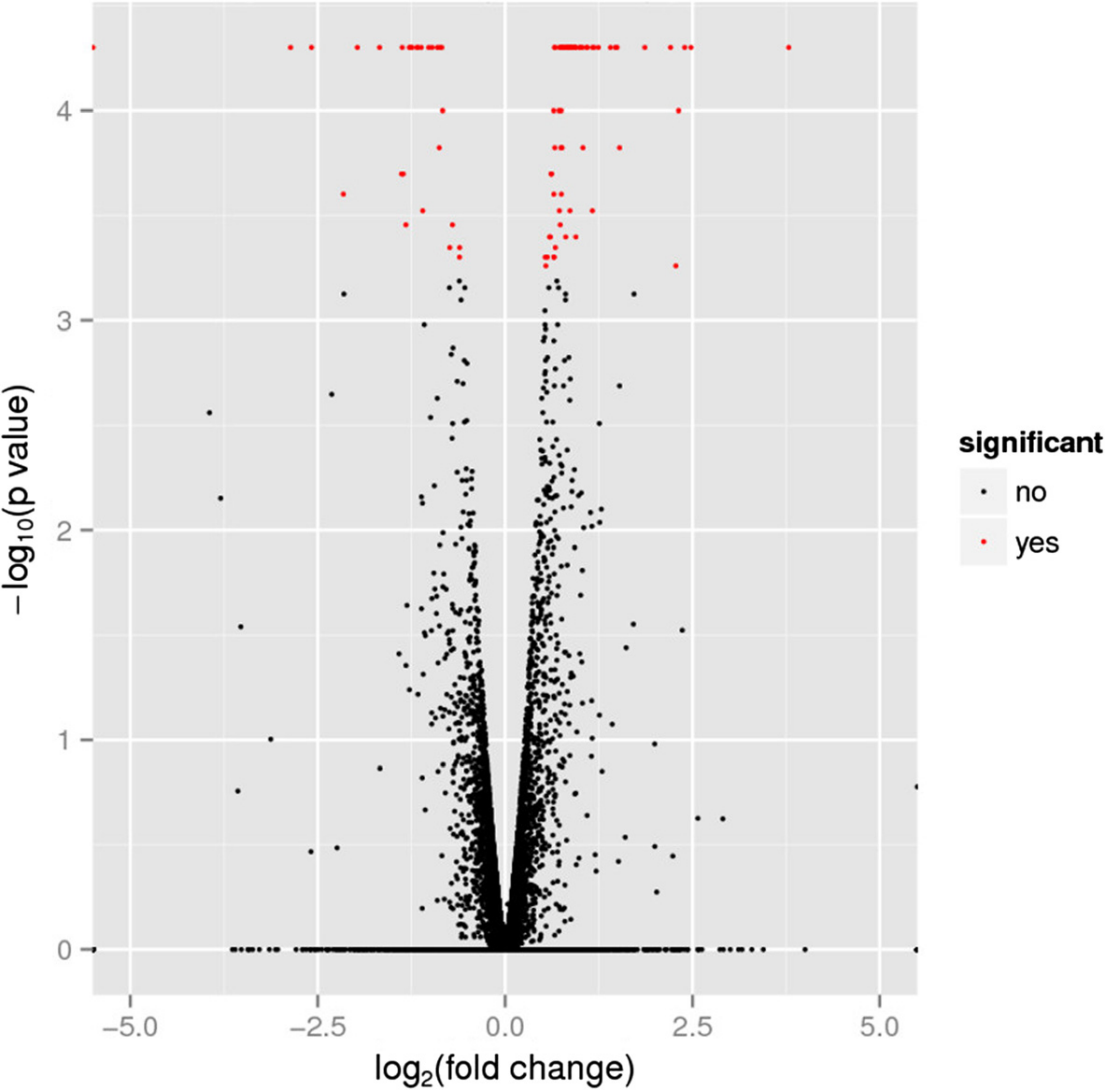
Volcano plot for honeybee data set. Volcano plot for the 11169 genes from the honeybee data. The x-axis is the fold-change value and the y axis is the - log10 p-value. Using the p-value 0.05 as the threshold cutoff, 96 genes in the upper left and upper right are selected. Red spots indicate the statistically significant DEGs

From the 11 168 genes referenced in the genome of *A. mellifera*, 10 519 genes were found to be expressed in the hygienic pools and 10 374 genes in the non-hygienic pools. The top 10 expressed genes were the same for the two behavioral conditions, but their order differed (Table 3). All are genes involved in royal jelly production. The other major royal jelly protein genes (Mrjp) were also detected in our data, but at lower levels. Ninety-six genes were found differentially expressed between hygienic and non-hygienic bees (Fig. 1). Twenty-eight genes were over-expressed in hygienic bees and 17 of these had a log2 fold change higher than 1, meaning that expression of the gene was two times or higher in hygienic bees (Table 4). The three most DEGs (log2 fold change > 2) were CYP6AS1, Syn1 and LOC100577331. LOC727589 was appended to this list because it was not expressed in non-hygienic bees but lightly expressed in all hygienic colonies. Mir375 and Mir252 genes showed relevant patterns but, as they were not expressed in all hygienic colonies, were not statistically significant. These two genes were highly expressed in two colonies, 571 and 586 respectively. Sixty-eight genes were over-expressed in non-hygienic bees (Table 5), with a fold change higher than 1 for 20 of them. Six genes were highly differentially expressed: Hex70c, LOC410988, LOC552229, LOC100576440, LOC726319, LOC727570.

**Table 3:** Top 10 expressed genes of *Apis mellifera* transcriptome

Gene	Chromosome	Hygienic	Non-hygienic	Annotation/product
LOC406093	Chr6	309432	411862	Apisimin
Mrjp1	Chr11	147010	184154	Major royal jelly protein 1
Mrjp3	Chr11	39892.9	57788.7	Major royal jelly protein 3
LOC551813	Unplaced scaffold	37710.2	41357.9	Major royal jelly protein 1-like
Mrjp2	Chr11	26601.4	32467.6	Major royal jelly protein 2
LOC727045	Unplaced scaffold	18235.8	15339.8	Major royal jelly protein 3-like
Mrjp5	Chr11	17599.8	20420.4	Major royal jelly protein 5
Mrjp7	Chr11	11319.4	12935.5	Major royal jelly protein 7
LOC406081	Chr5	10954.5	11105.2	Glucose oxidase
Mrjp4	Chr11	9981.09	13858	Major royal jelly protein 4

**Table 4:** List of genes significantly over-expressed in hygienic honeybees

Gene	Chromosome	Hygienic FPKM	Non-hygienic FPKM	q_value	Gene product
Cac	Chr3	7.79884	5.12143	0.0435185	cacophony
CPR5	Chr16	72.64	43.5375	0.0435185	cuticular protein 5
CYP6AS1	Chr13	2.04404	0.281032	0.00755	cytochrome P450 6AS1
Gat-a	Chr1	11.793	6.29334	0.00755	GABA neurotransmitter transporter-1A
LOC100576698	Unplaced scaffold	21.7757	11.0732	0.00755	hypothetical LOC100576698
LOC100576840	Chr12	23.4989	9.15222	0.0239083	hypothetical protein LOC100576840
LOC100577331	Chr10	39.1592	8.77503	0.02869	hypothetical protein LOC100577331
LOC100578672	Chr4	4.01496	1.02343	0.00755	hypothetical protein LOC100578672
LOC100578804	Chr6	2.30117	0.888719	0.00755	hypothetical LOC100578804
LOC408734	Chr3	6.43168	2.8719	0.00755	succinate dehydrogenase
LOC410149	Chr12	217.491	122.044	0.0138823	plasma glutamate carboxypeptidase-like
LOC410207	Chr10	2.67182	1.459	0.00755	dihydropyrimidine dehydrogenase
LOC410689	Chr1	18.0555	11.8371	0.0457819	hypothetical protein LOC410479
LOC412162	Chr7	1.65605	0.661079	0.0367372	armadillo repeat-containing protein 4-like
LOC551908	Chr15	2.32533	0.728909	0.00755	hypothetical protein LOC551908
LOC552190	Chr12	8.46323	5.19949	0.0367372	hypothetical LOC552190
LOC552369	Unplaced scaffold	5.3599	2.915	0.018986	synaptotagmin-like protein 5-like
LOC552388	Unplaced scaffold	12.1403	5.06737	0.00755	major royal jelly protein 1-like
LOC552604	Unplaced scaffold	2.69267	1.2569	0.0326848	SLIT-ROBO Rho GTPase-activating protein 1-like
LOC724228	Chr5	1.43709	0.551194	0.0239083	neprilysin-2-like
LOC724749	Chr7	1.18011	0.499495	0.00755	hypothetical protein LOC724749
LOC725449	Unplaced scaffold	60.6323	26.79	0.00755	hypothetical protein LOC725449
LOC725646	Chr4	313.389	154.585	0.00755	n-acetylneuraminate lyase-like
LOC727589	Unplaced scaffold	1.75295	0	0.00755	rab3 GTPase-activating protein catalytic subunit-like
Myo20	Chr10	2.27885	0.939329	0.00755	myosin 20
Notum	Chr1	3.94958	1.81886	0.00755	notum pectinacetylesterase homolog
Pka-C1	Chr2	5.37177	2.98956	0.00755	cAMP-dependent protein kinase 1
Syn1	Chr10	23.5363	3.92432	0.00755	syntrophin-like 1

**Table 5:** List of genes significantly over-expressed in non hygienic honeybees

Gene	Chromosome	Hygienic FPKM	Non-hygienic FPKM	q_value	Gene product
A4	Chr2	154.151	285.535	0.00755	apolipophorin-III-like protein
bgm	Chr1	10.1512	16.0742	0.018986	very long-chain-fatty-acid--CoA ligase bubblegum
Cda5	Chr10	3.65306	5.76859	0.00755	chitin deacetylase-like 5
CTL6	Chr11	2.9341	4.60572	0.02869	C-type lectin 6
CYP4AZ1	Chr11	3.8045	7.78363	0.00755	cytochrome P450 4AZ1
Cyp4g11	Chr16	6.38739	11.5389	0.00755	cytochrome P450 4G11
CYP6AS11	Chr13	3.04052	4.78082	0.0457819	cytochrome P450 6AS11
CYP6AS8	Chr13	41.8902	93.879	0.00755	cytochrome P450 6AS8
FAR1	Chr12	30.999	56.0177	0.00755	fatty acyl-CoA reductase 1
GMCOX12	Chr1	1.61714	3.46505	0.00755	GMC oxidoreductase 12
Grx-like1	Chr6	0.861762	1.72838	0.00755	glutaredoxin-like 1
Hex70c	Chr8	0.137575	1.897	0.00755	hexamerin 70c
jhamt	Chr4	7.73278	12.7786	0.0326848	juvenile hormone acid methyltransferase
LOC100576118	Chr2	4.8343	8.50859	0.00755	hypothetical protein LOC100576118
LOC100576440	Unplaced scaffold	1.1984	5.9613	0.0138823	probable cytochrome P450 6a13-like
LOC100577133	Chr9	22.7738	37.9257	0.0367372	hypothetical LOC100577133
LOC100577380	Chr7	53.7547	107.429	0.00755	protein takeout-like
LOC100577883	Unplaced scaffold	25.0911	46.3119	0.00755	probable cytochrome P450 4aa1-like
LOC100578120	Chr11	21.2648	35.1245	0.00755	hypothetical protein LOC100578120
LOC406105	Chr14	50.4087	106.872	0.00755	esterase A2
LOC406144	Chr10	68.5429	153.668	0.0326848	abaecin
LOC408361	Chr11	23.3868	39.3707	0.02869	alpha-tocopherol transfer protein-like
LOC408414	Chr13	16.1569	28.3998	0.00755	tropomyosin-1-like
LOC408420	Chr13	2.60398	4.08972	0.0457819	RING finger protein nhl-1-like
LOC408474	Chr14	5.19202	8.53786	0.0138823	apyrase
LOC408608	Chr1	27.8681	101.414	0.00755	hypothetical LOC408608
LOC409060	Chr5	11.8421	20.277	0.00755	hypothetical LOC409060
LOC409740	Chr15	12.4087	21.2398	0.00755	clavesin-1-like
LOC409787	Chr6	18.342	35.0628	0.00755	paramyosin
LOC410087	Chr2	42.6442	62.1185	0.0493109	protein lethal
LOC410736	Chr1	0.794104	1.39138	0.0400326	ELAV-like protein 2-like
LOC410788	Chr1	0.448281	1.06158	0.00755	NMDA kainate sensitive receptor
LOC410988	Unplaced scaffold	0.686025	3.82988	0.00755	acyl-CoA synthetase family member 2
LOC411202	Chr4	9.15592	16.741	0.00755	alcohol dehydrogenase
LOC411285	Chr8	19.9416	33.524	0.0138823	muscle LIM protein Mlp84B-like
LOC413907	Chr1	0.924643	1.89985	0.018986	hypothetical protein LOC413907
LOC413936	Chr4	1.7179	3.04752	0.00755	hypothetical protein LOC413936
LOC551407	Unplaced scaffold	1.72238	4.56605	0.00755	A disintegrin and metalloproteinase with thrombospondin motifs 14-like
LOC551761	Chr1	2.2724	4.36878	0.00755	alpha-tocopherol transfer protein-like
LOC552149	Chr3	11.4936	20.8411	0.00755	aquaporin AQPAn.G-like
LOC552229	Chr1	0.483327	2.54394	0.00755	esterase B1-like
LOC552598	Unplaced scaffold	1.15471	2.22152	0.0400326	hypothetical LOC552598
LOC724239	Chr1	22.1989	61.5634	0.00755	kynurenine
LOC724644	Chr13	19.3289	32.6522	0.00755	hypothetical protein LOC724644
LOC725017	Chr6	12.7717	29.0823	0.00755	UDP-glycosyltransferase
LOC725026	Chr9	4.35135	8.17989	0.00755	retinol dehydrogenase 10-A-like
LOC725051	Chr11	7.28613	13.7766	0.00755	probable multidrug resistance-associated protein lethal
LOC725238	Chr1	26.8078	39.6372	0.0457819	hypothetical protein LOC725238
LOC725413	Unplaced scaffold	0.605573	1.10389	0.0326848	fibrillin-2-like
LOC725838	Chr8	2.51888	7.09723	0.00755	hypothetical protein LOC725838
LOC726040	Chr13	14.3708	21.9656	0.0239083	probable 4-coumarate--CoA ligase 3-like
LOC726319	Unplaced scaffold	0.308448	1.4963	0.0493109	hypothetical protein LOC726319
LOC726418	Chr16	8.43603	12.8232	0.0400326	flavin-containing monooxygenase FMO GS-OX-like 3-like
LOC726672	Chr6	3.45246	6.24059	0.00755	hypothetical protein LOC726672
LOC726733	Chr4	10.3501	16.2209	0.0138823	cysteine-rich protein 1-like
LOC726790	Chr13	5.8331	8.4475	0.0457819	hypothetical protein LOC726790
LOC727202	Unplaced scaffold	1.03791	2.99235	0.018986	carbohydrate sulfotransferase 8-like
LOC727570	Unplaced scaffold	1.85047	8.55136	0.00755	CD63 antigen-like
MsrA	Chr1	23.8646	35.9934	0.0400326	methionine sulphoxide reductase A
ND1	ChrMT	32.2564	49.6	0.0239083	NADH dehydrogenase subunit 1
ND4	ChrMT	29.1365	46.2524	0.00755	NADH dehydrogenase subunit 4
NimC2	Chr15	4.3505	6.92319	0.0435185	nimrod C2
Obp4	Chr9	18.3146	31.0485	0.018986	odorant binding protein 4
SP23	Chr4	4.28102	7.14745	0.00755	serine protease 23
Sur	Chr3	0.985398	1.64945	0.018986	sulfonylurea receptor
TpnCIIIa	Chr12	46.8412	79.459	0.00755	troponin C type IIIa
TpnI	Chr2	31.1182	54.9751	0.00755	troponin I
TpnT	Chr6	30.7717	59.0756	0.00755	troponin T

**Fig. 2:**
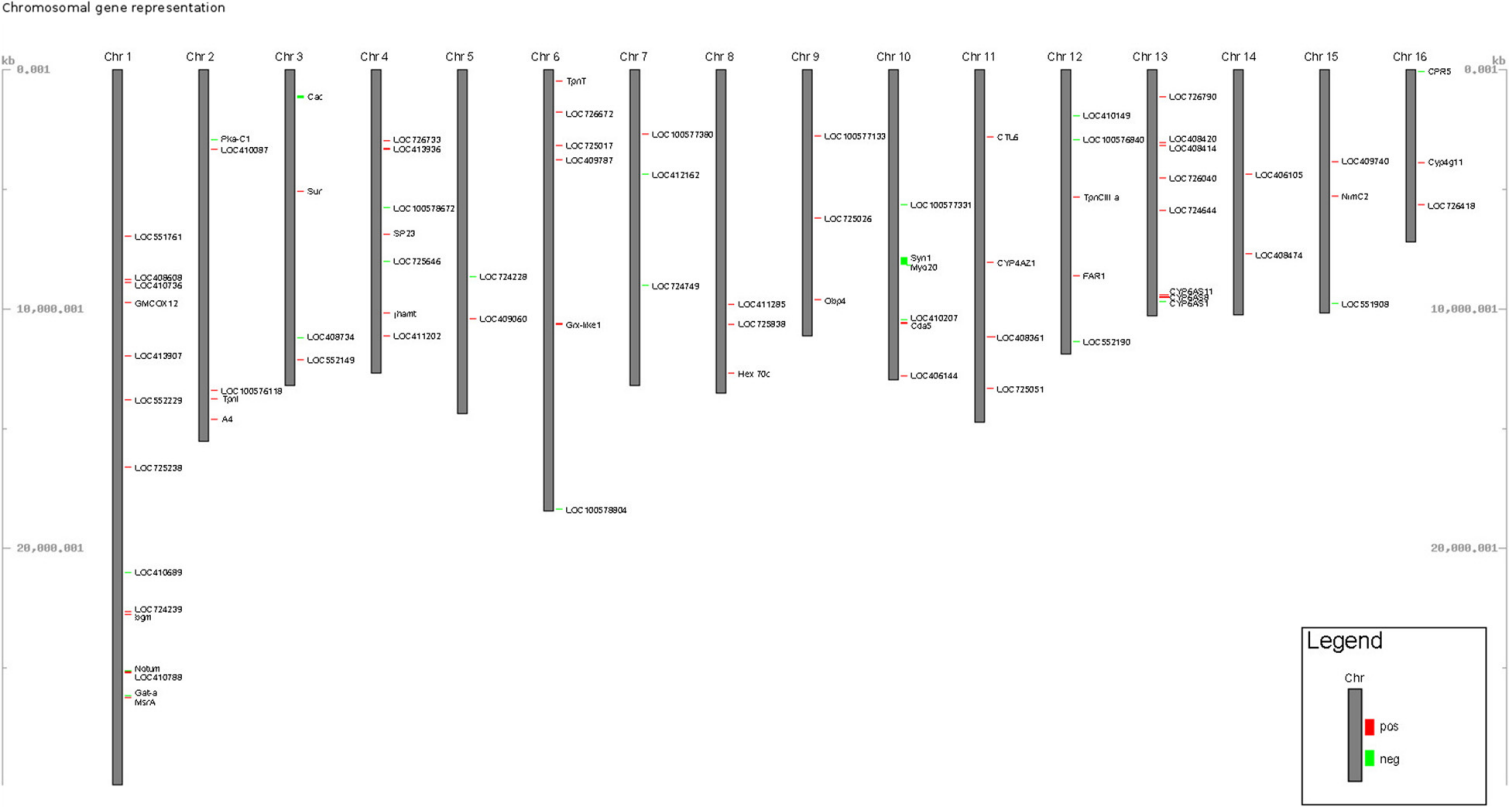
Chromosomal position of the DEGs on the *Apis mellifera* genome. Red spots indicate a gene over-expressed on non-hygienic bees and; green spots indicate an under-expression in non-hygienic bees

Among the 96 DEGs, 79 were located on all the 16 linkage groups of the honeybee genome, 2 on the mitochondrial chromosome (ND1 and ND4) and 15 on unplaced scaffolds (Fig. 2). Two of the three genes highly related to hygienic behavior were located on chromosome 10 (Syn1 and LOC100577331) and the third one on chromosome 13 (CYP6AS1). Concerning the non-hygienic genes, only Hex70c and LOC552229 were located on chromosomes 8 and 1 respectively. The other genes were located on four different unplaced scaffolds.

Genetic links with hygienic behavior have been studied previously in order to detect QTLs associated to this behavior [30–33]. By comparing our results to accessible previous data (Table 6), the genomic sequences of 22 gene candidates (i.e. exhibiting DGE) were located inside the confidence interval of 95 % of all the QTLs influencing hygienic behavior (Table 6).

**Table 6:** Comparison of the DEGs found in transcriptome analysis with data from previous QTLs studies. Genes in red are over-expressed in non hygienic bees; green represents the over-expressed genes in non hygienic bees

Study	QTL	Chromosome	Nearest marker	Nearest gene
Oxley *et al.* 2010	Uncap1	9	AT128	Obp4
Oxley *et al.* 2010	Uncap2	16	K1601	CPR5
Oxley *et al.* 2010	Rem1	10	AC074	Syn1, Myo20
Oxley *et al.* 2010	hyg 1	2	K0263	A4
Oxley *et al.* 2010	hyg 2	5	A0058	
Oxley *et al.* 2010	hyg 3	16	K1601	CPR5
Tsuruda et al. 2012		1		
Tsuruda et al. 2012		9	9224292	Obp4
Spotter *et al.* 2012	LG1:3039231-8453574	1		
Spotter *et al.* 2012	LG1:9418717-16819942	1		GMCOX12, LOC413907, LOC552229, LOC725238
Spotter *et al.* 2012	LG2:1-12503099	2		Pka-C1, LOC410087
Spotter *et al.* 2012	LG6:11206828-17739083	6		LOC726672
Spotter *et al.* 2012	LG7:9515998-12848973	7		
Spotter *et al.* 2012	LG12:1-4003353	12		LOC410149, LOC100576840
Spotter *et al.* 2012	LG13:5247545-10266737	13		LOC724644, CYP6AS11, CYP6AS8, CYP6AS1
Spotter *et al.* 2012	LG15:1-6643609	15		LOC409740, NimC2
Spotter *et al.* 2012	LG16:3196393-6242592	16		Cyp4g11, LOC726418

**Fig. 3:**
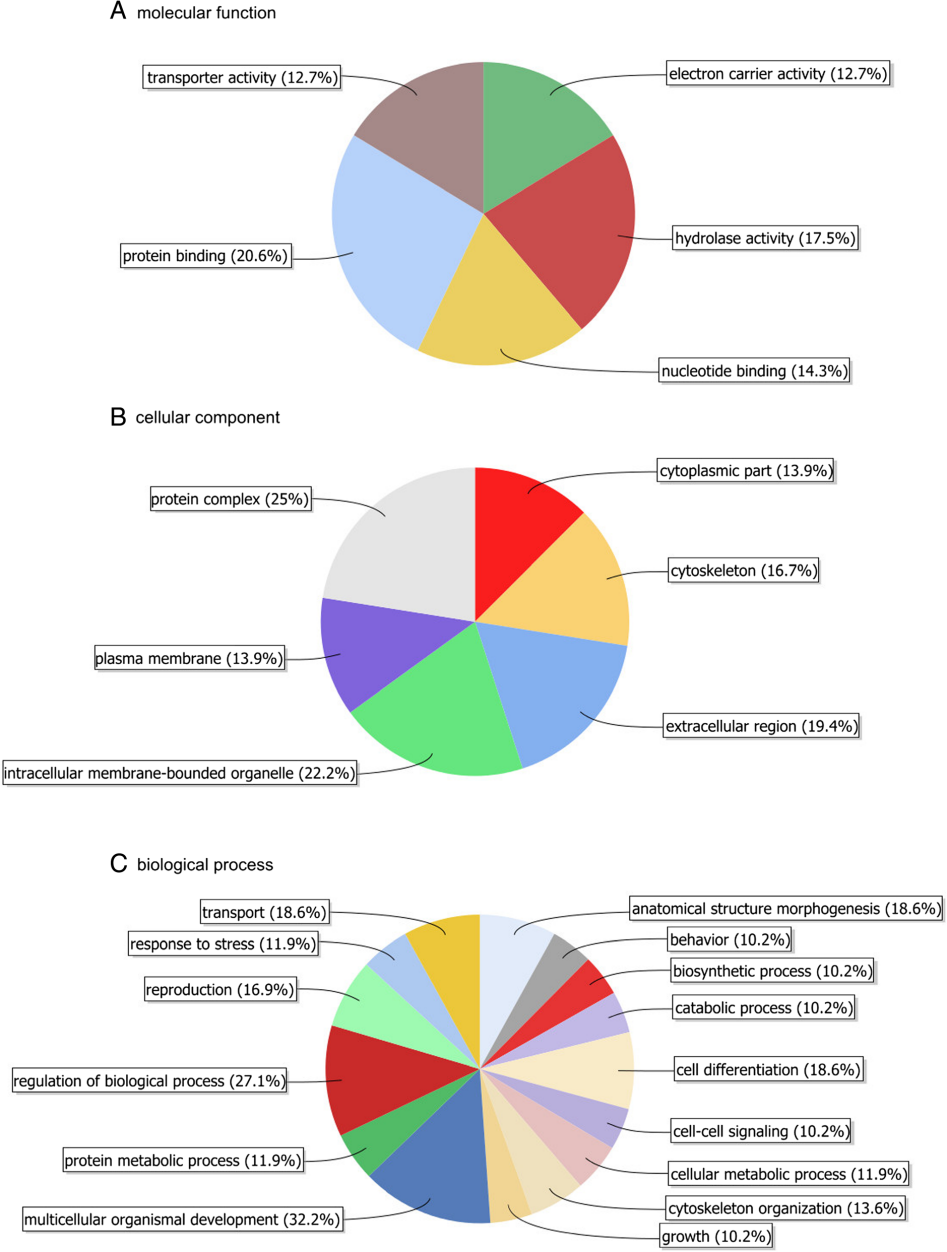
Multi-level pie chart of the major GO-categories represented in the DEG dataset. Panel a represented the GOterm associated to molecular function, Panel b represented the GO-term associated to cellular component andPanel c represented the GO-term associated to biological process

For the 96 genes differentially expressed, 71 were associated with at least 1 GO-term and 86 with an interproscan result. The ontology covered three domains: cellular component, molecular function and biological process. Twenty-four GO-terms classified in molecular function were found and were recovered by 63 genes (Fig. 3a and Table S1). For the cellular component domain, 36 genes were assigned to 17 GO-terms (Fig. 3b and Table S2). Fifty-nine genes were assigned to a biological process (41 GO-terms) (Fig. 3c and Table S3). Furthermore, 15 DGEs were assigned as potential transcription factors by direct blast to a drosophila gene belonging to the GO-term DNA binding or by the Blast2Go annotation with the GO-terms DNA binding or Nucleotide binding. For the KEGG pathways analysis, 10 genes were involved in 13 KEGG pathways. KEGG pathways and the enzyme involved are presented in Table 7.

**Table 7:** KEGG pathways analysis of the DEGs found in this study. Genes in red font are over-expressed in non hygienic bees; green represents the over-expressed genes in non hygienic bees

Pathways	Enzymes	Genes
Purine Metabolism	ec:3.1.3.5- uridine 5’-nucleotidase; ec:3.6.1.3- adenylpyrophosphatase	LOC408474; LOC725051,Sur
Oxidative phosphorylation	ec:1.6.5.3 - reductase (H + −translocating); ec:1.3.5.1-deshydrogenase (ubiquinone)	ND4; LOC408734
Glycolysis/Gluconeogenesis	ec:1.1.1.2-dehydrogenase (NADP+)	LOC411202
Propanoate metabolism	ec:6.2.1.4-ligase (GDP-forming)	LOC408734
Caprolactam degradation	ec:1.1.1.2-dehydrogenase (NADP+)	LOC411202
Glycerolipid metabolism	ec:1.1.1.2-dehydrogenase (NADP+)	LOC411202
Pyrimidine metabolism	ec:3.1.3.5- uridine 5’-nucleotidase	LOC408474
Citrate cycle (TCA cycle)	ec:6.2.1.4-ligase (GDP-forming); ec:1.3.5.1-deshydrogenase (ubiquinone)	LOC408734
Glycine, serine and threonine metabolism	ec:1.1.99.1-dehydrogenase	GMCOX12
Amino sugar and nucleotide sugar metabolism	ec:3.5.1.41-deacetylase	Cda5
Nicotinate and nicotinamide metabolism	ec:3.1.3.5- uridine 5’-nucleotidase	LOC408474
mTOR signaling pathway	ec:2.7.11.11-protein kinase	Pka-C1
Fatty acid degradation	ec:6.2.1.3-ligase	bgm

Gene set enrichment was performed to compare the enrichment of the different GO-terms between the 96 genes differentially expressed and the whole genome. The results of this analysis indicate that GO-term electron carrier activity (GO-ID: GO:0009055) is over-represented in our DEGs. The genes involved in this GO-term were: CYP4AZ1, CYP6AS8, CYP6AS1, CYP6AS11, LOC100576440, Cyp4g11 and LOC100577883. All of these genes were involved or considered as potentially involved in the cytochrome P450. Six of these genes were over-expressed in non-hygienic bees, and only gene CYP6AS1 was over-expressed in hygienic bees.

## Discussion

To our knowledge, this study is the first to correlate honeybee hygienic behavior with differential gene expression. Almost all previous studies that analyzed honeybee RNA focused on the parasitic / pathogenic reaction and its variability among different honeybee castes [30, 33, 39–41]. In this study, we used RNA-seq to highlight, at the genomic scale, which genes are differentially expressed in hygienic versus non-hygienic colonies.

Our data indicates that not all previously referenced genes present in the genome of honeybee are expressed in our bee brain samples. Only 10 519 genes (94.2 % of all genes referenced) were found expressed in hygienic honeybees and 10 374 genes (92.9 % of all genes referenced) in non-hygienic bees. Genes may be missing from our samples due to the fact that we studied gene expression only for the RNA pools from bee brains, and not the entire tissue composites. It has previously been demonstrated that tissue specificity is highly important for RNA-seq design [42].

The distribution of gene expression was very similar between both behavioral states (hygienic and non-hygienic). This result can be explained by the fact that the most abundantly expressed genes were not differentially expressed (Table 3). Interestingly, the genes most expressed in our samples were all involved in processing royal jelly. This finding is consistent with a previous study that found nurses and foragers highly expressed the Mrjp gene family [40]. As in the article of Liu *et al.* 2011, we found a low level of expression of the Mrjp 6 in bee brains.

The differences between the two behavioral states rely on few genes (96 genes), which is 94 % less than differences between nurses and foragers (1621 genes with a fold change greater than 2) [40, 41]. However, this finding is consistent with results between such less-differentiated castes as guard, undertaker or comb builder [41]. It seems that, as for other task specializations (comb building, guarding and undertaking), few genes influence the performance of hygienic behavior, and they are thus more tightly regulated than caste specialization. This could be explained by the fact that caste specialization (queen, nurse, forager) is strongly influenced by the environment (queens vs workers) or age-related differences [43–45]. The low numbers of genes differentially expressed (DEGs) between the two intra-caste behavioral states may reflect a difference in bee age. Consequently, as the difference in behavioral state is the only variable among our samples, these few DEGs relate to differing behavioral performance that occurs according to a design independent of any age limit or caste. Furthermore, we hypothesize that expression of gene involved in the hygienic behavior is constitutive and not facultative, especially during the detection of diseased brood [20]. This ensures that the differential expression of these genes is related to the hygienic behavior and not to age or caste. This assumption is also supported by the fact that RNA analysis was performed on a pool of 25 nurses so it represented the global transcription of the caste and not an individual gene expression event.

All DEGs are statistically significant (FDR correction, q-value < 0.05) despite the fact that fold-changes are at low levels. Low level fold-change acts in favor of a subtle modification of brain gene expression, much as has been suggested for task specialization [41]. However, if we consider the higher level of fold-change, gene dispersion is concentrated on unplaced scaffolds (4 genes) and chromosomes 1, 8, 10 and 13 (respectively 1, 1, 2 and 1 genes). Interestingly, chromosomes 1, 10 and 13 are known to carry QTLs linked to honeybee hygienic behavior [30, 31, 33]. This result is also supported by the fact that genomic sequences of the 22 DEGs are located in the confidence interval of all the QTLs influencing hygienic behavior (Table 6) except for the QTL named *hyg 2*, located on chromosome 5, the QTL region localized on chromosome 7 by Spötter *et al.* and the QTL found in chromosome 1 by Tsuruda *et al.* 2012. These results show that the DEGs found in our study are consistent with the previous literature. However, it also seems that regulation of the gene expression linked to hygienic behavior is spread more widely throughout the genome than previously thought. The localization of these genes is, however, quite surprising, because it was thought that the genetic basis of hygienic behavior was localized on few loci. We unexpectedly found DEGs on all 16 honeybee chromosomes, as well as on mitochondrial chromosomes and unplaced scaffolds. These results suggest a wider regulation of the transcription. The high number of DGEs classified as potential transcription factors supports this supposition. Four of them (GMCOX12, LOC725238, Myo20 and Pka-C1) are even located on hygienic QTL positions (chromosomes 1, 1, 10 and 2 respectively).

Gene Ontology analysis shows that the two biological processes most represented by the DEG are multicellular organismal development and regulation of biological processes. This result indicates that most DEGs contribute to the development of larvae into adult bees and are also involved in gene expression regulation, protein modification or interaction with a protein or substrate molecule. These two biological processes are consistent with the theory that few genes have a wide influence (as transcription factors). At the level of molecular function, the three most represented GO-terms are protein binding, hydrolase activity and nucleotide binding. As previously discussed, protein binding and nucleotide binding are functions that are involved in the regulation of molecular processes like transcription. It seems that hygienic and non-hygienic bees differ in their transcription pathways. Interestingly, hydrolase activity is particularly over-expressed in non-hygienic bees. Hydrolase activity is a process involved in the catalysis of various bonds, including the catalysis of peptides such as the one that can signal the presence of diseased brood.

Gene Set Enrichment analysis of the DEG shows that only one GO-term is over-represented (FDR correction, q-value < 0.05): electron carrier activity (GO-ID: GO:0009055). Furthermore, all the genes associated with this GO-term are involved or can be considered as potentially involved in cytochrome P450 pathway. Among these genes, some are coding for different enzymes of the cytochrome P450. These enzymes are known to be involved in many processes, particularly detoxification of xenobiotics and hormonal degradation. Furthermore, they are suspected of playing a role in the degradation of odorants, pheromones or defensive chemicals [46]. The threshold for detection of diseased brood is one key factor in how quickly a nurse can detect and initiate the removal process. This detection capacity could be influenced by the nurse’s olfactory capabilities [34–36]. We can therefore hypothesize that non-hygienic bees that over-express cytochrome P450 enzymes degrade the odorant pheromones or chemicals that normally signal the presence of diseased brood before activation of the removal process. The bees are then less efficient in detecting contaminated broods. The high level of cytochrome P450 enzymes in non-hygienic bees can be explained by two non-exclusive hypotheses. First, the non-hygienic bees may have a constitutively higher expression of these enzymes due to the differences observed in regulation patterns, as previously discussed. Or, the induction of cytochrome P450 gene expression may be due to a higher sensitivity to xenobiotics. It has been demonstrated that xenobiotics can enhance the expression of cytochrome P450 genes [47]. A higher sensitivity to xenobiotics could then induce a stronger response by over-expressing cytochrome P450 genes, which in turn might alter the performance of hygienic behavior.

## Conclusions

This study is the first to characterize the transcriptomic basis of the differential performance of hygienic behavior by the honeybee (*Apis mellifera L.*). Our findings show that hygienic behavior relies on a limited set of genes, most collocated with the QTLs described in previous studies as playing major roles in honeybee hygienic behavior. The differences between behavioral states (hygienic and non-hygienic) can be explained by different regulation patterns (expression level and biological processes) associated with an over-expression of cytochrome P450 genes. These candidate genes provide relevant targets for SNPs analysis (cis-regulatory sites and coding sequence) to develop molecular tools for honeybee genetic programs, which would provide a rapid and efficient method for selecting honeybee colonies with a high level of hygienic behavior.

## Methods

### Ethics statement

The owner of the land on which the hives were located gave permission to conduct the study on the site. No additional permit was required, considering the fact that the owner gave his permission. The GPS coordinates were (46°40’31.06” N 71°54’57.98” W). The field study did not involve endangered or protected species.

### Sample collection

This study was based on 13 managed honeybee colonies (*Apis mellifera*), selected from among the livestock of our bee research facility (Deschambault, Québec, Canada) in June 2012. Young queens had been introduced in these colonies in July 2011. These queens were hybrid Italian/Buckfast stock obtained through our selection program. Each colony was comprised of a 9-frame Langstroth hive body. Selected colonies were all of equivalent strength (6–7 frames of bees/brood). All hives were placed in the same apiary to avoid influence of environmental conditions. The freeze-killed brood assay [38] was chosen to measure the hygienic behavior capability of each colony. This test consisted of freezing a patch of a pupated sealed brood with liquid nitrogen. Briefly, 100 mL of liquid nitrogen was poured on two circles (15 cm diameter) within the brood area of each hive (7 day old larvae). The liquid nitrogen was confined to a specific spot on the brood frame, covering an area of 60 cells. Hygienic behavior was evaluated by calculating the number of brood removed in a period of 24 h. The hygienic behavior of each hive was estimated as a percentage of the dead larvae removed by the worker bees. A colony that removed 90 % of the dead larvae or more was considered as hygienic and a colony that removed less than 60 % of the dead cells was considered as non-hygienic. Among the studied colonies, five colonies removed between (50–90) % of the cells; these were classified as intermediate, and were sequenced but not included in the differentially expressed genes (DEG) analysis. Forager bees can revert to brood task such as removing dead brood when there is a nectar flow. In our study, hygienic test was done in absence of a nectar flow (mid May). Honeybee samples were taken the day following the hygienic test and great care was taken to sample only bees that were on the brood frame where the hygienic test was performed.

### RNA extraction

Total RNA was extracted from a pool of 25 honeybee brains using TRIzol® Reagent protocol from Invitrogen [48] with some modifications. The 25 honeybee brains sampled from each colony were dissected and treated as one pool, then added separately to 1 mL Trizol with 50 mg of acid washed glass beads and gently mixed for 5 min. Samples were then incubated at room temperature for 5 min. 200 μL of Chloroform was added, mixed vigorously and the mixture was incubated at room temperature for 12 min (with a vortex step at mid-incubation) followed by a centrifugation at 12 000 g for 15 min at 4 ° C. Supernatant was then washed with 250 μL each of Isopropanol and hypersaline solution (1.2 M sodium citrate; 0.8 M NaCl) with incubation for 10 min at room temperature followed by centrifugation at 12 000 g for 15 min at 25 °C. The RNA pellet was subsequently washed twice with 1 mL 75 % ethanol and centrifuged at 12 000 g for 7 min at 24 °C. The pellet was dried and 30 μL of nuclease-free water was added to each extraction. Purity and quality of the RNA was assessed by quantification with a Nanodrop 2000 spectrophotometer (Thermo Scientific). Tubes were then stored at −80 °C.

### Library construction

To construct a paired-end library for Illumina sequencing, we used the Illumina TruSeq^TM^ RNA sample preparation kit according to the manufacturer’s instructions. First, sample quality was confirmed by an Experion RNA analysis following the Experion RNA StdSens analysis kit protocol (Bio-Rad). Then, 4 μg of the total RNA sample was used for poly-A mRNA selection using streptavidin-coated magnetic beads. This protocol uses two rounds of enrichment for poly-A mRNA followed by mRNA fragmentation. The fragmented mRNA samples were subjected to cDNA synthesis using the Illumina TruSeq^TM^ RNA sample preparation kit according to the manufacturer’s protocol. Briefly, cDNA was synthesized using reverse transcriptase (Super-Script II) and random primers. The cDNA was further converted into double stranded DNA using the reagents supplied in the kit, and the resulting dsDNA was used for library preparation. The double-stranded cDNA obtained was subjected to library preparation using the Illumina TruSeq™ RNA sample preparation kit (Low-Throughput protocol) according to the manufacturer’s protocol. The quality of the library was controlled on an Agilent technologies 2100 bioanalyser following the protocol provided with the Agilent DNA 7500 kit. In the final step before sequencing, all 13 individual libraries were normalized and pooled together using the adapter indices supplied by the manufacturer (Illumina indexes 3–8, 12–16, 18, 19). Pooled sequencing was then performed as 150 bp, paired-end reads in a single lane of an Illumina HiSeq2000 instrument at McGill University and the Génome Québec Innovation Centre.

### Data processing

The raw reads were first assessed and trimmed for quality using the FASTX toolkit (http://hannonlab.cshl.edu/fastx_toolkit/). Reads with a bad quality score (phred score < 20) were discarded. The remaining reads were synchronized, and single reads were also discarded. Reads were then assembled and mapped to the reference genome of the honeybee (ftp://ftp.ncbi.nlm.nih.gov/genomes/Apis_mellifera/) using Tophat [49, 50]. Reads were assembled for different K-mer values (Kmer = 27 to Kmer = 53) to ensure good quality assembly. The data obtained were analyzed for the numbers of reads mapped, and the best quality mapping was selected with SAMStat [51].

### Identification of Differentially Expressed Genes (DEG)

Gene expression was calculated with Cufflinks [50] based on the honeybee genome as well as the annotation file downloaded from the NCBI database (ftp://ftp.ncbi.nlm.nih.gov/genomes/Apis_mellifera/GFF/). The abundance of each transcript mapped against the 11 169 genes annotated on the reference was estimated. The abundance of each transcript was then normalized by calculating the transcript abundance in Fragment Per Kilobase of exon per million fragments mapped (FPKM). Gene and transcript expression changes among samples were analyzed with Cuffdiff software. Differential expression was considered as statistically significant when the q-value (FDR correction) was lower than 0.05.

### Gene Ontology (GO) annotation

All transcripts of *Apis mellifera* referenced in the NCBI ftp server (ftp://ftp.ncbi.nlm.nih.gov/genomes/Apis_mellifera/RNA/) were used as a reference database. Genes were mapped to the references by a blast search (parameters: E-Value Hit Filter = 1e-06; Annotation cutoff = 55 and Go-weigth = 5) to retrieve the GO terms associated. Then query sequences from the pool of GO terms were assigned to functional terms. Mapping from GO terms to enzyme codes permits the subsequent recovery of enzyme codes and KEGG pathway annotations. Once this database was constructed, we performed the same analysis on the differentially expressed genes (DEG). GO enrichment analysis of functional significance maps all DEGs to terms in GO database. Then, a comparison of GO-term abundance between the differentially expressed gene set and the genome background was performed to look for enriched GO-terms. KEGG pathways enrichment analysis is based on the same procedure, so we compared the pathway representation in the DEG set and the genome background to identify significantly enriched metabolic pathways or signal transduction pathways. All of these analyses were performed with Blast2GO [52].
